# Supplementary figures and images for: Metatranscriptomic insights into the dengue patient blood microbiome: Enhanced microbial diversity and metabolic activity in severe patients
Source: PLoS Negl Trop Dis. 2024 Oct 17;18(10):e0012589. doi: 10.1371/journal.pntd.0012589 (PMC11521311; doi:10.1371/journal.pntd.0012589)

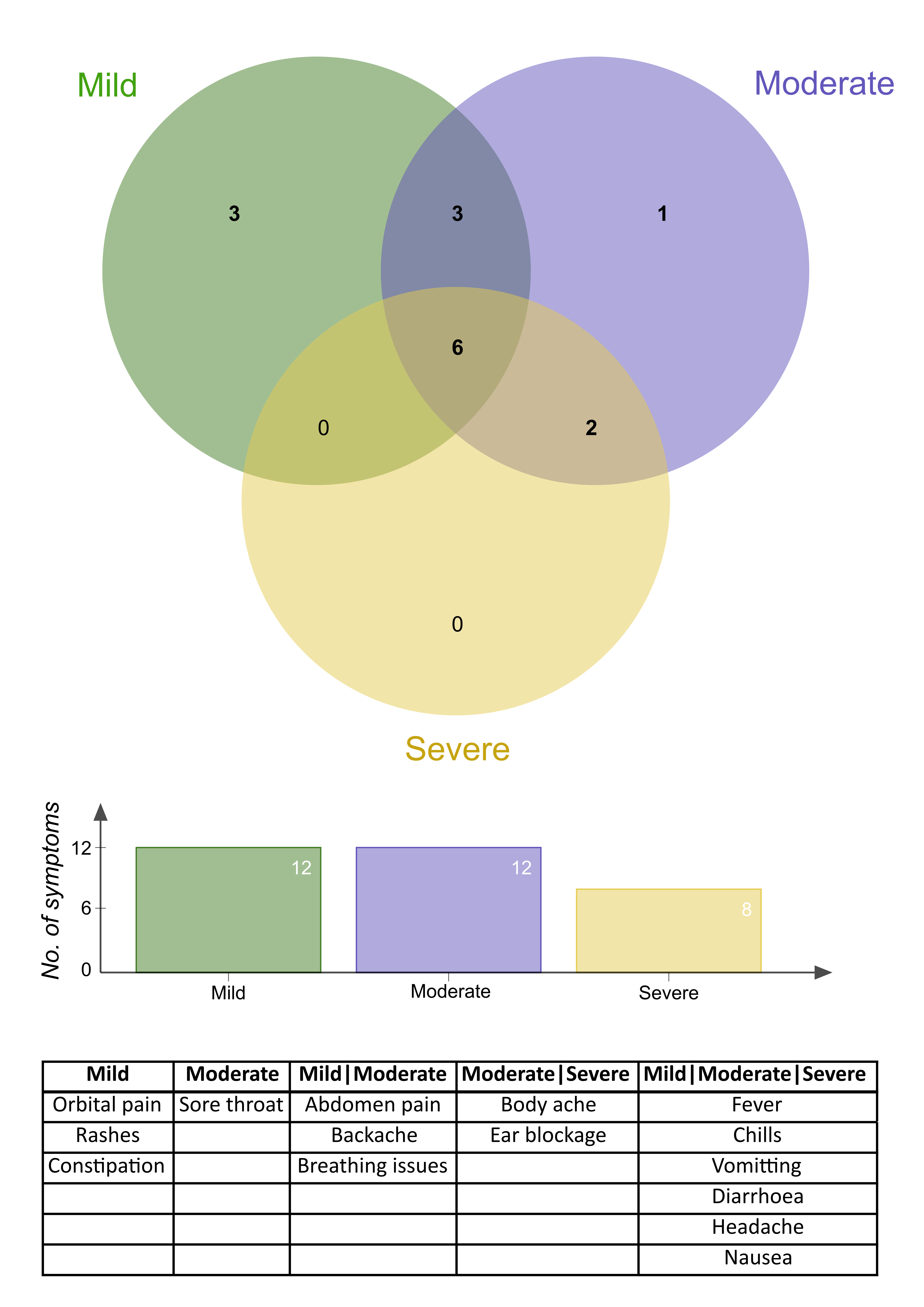

Supplement: S1 Fig — (PNG) [file pntd.0012589.s004.png]

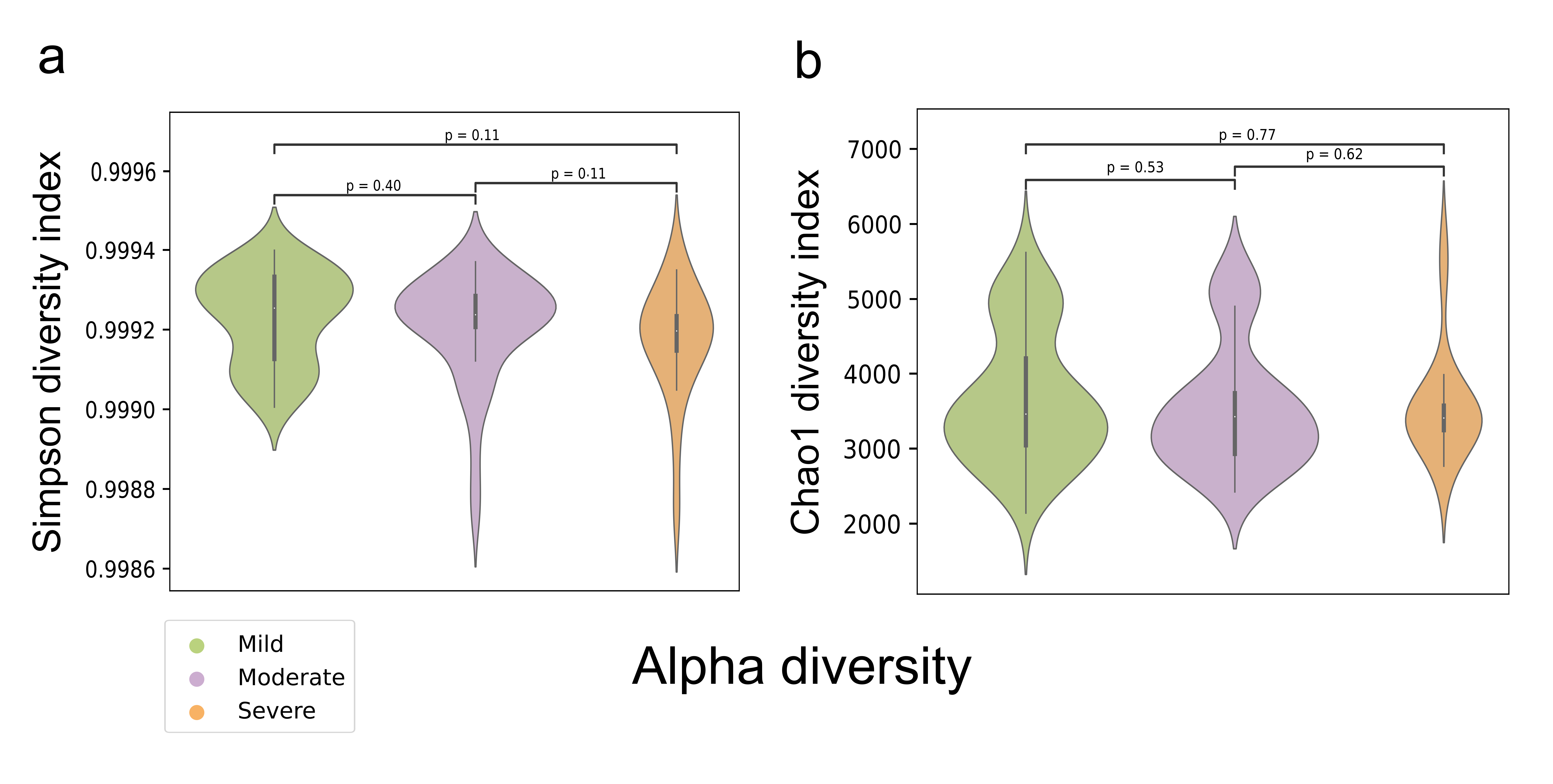

Supplement: S2 Fig — (TIF) [file pntd.0012589.s005.tif]

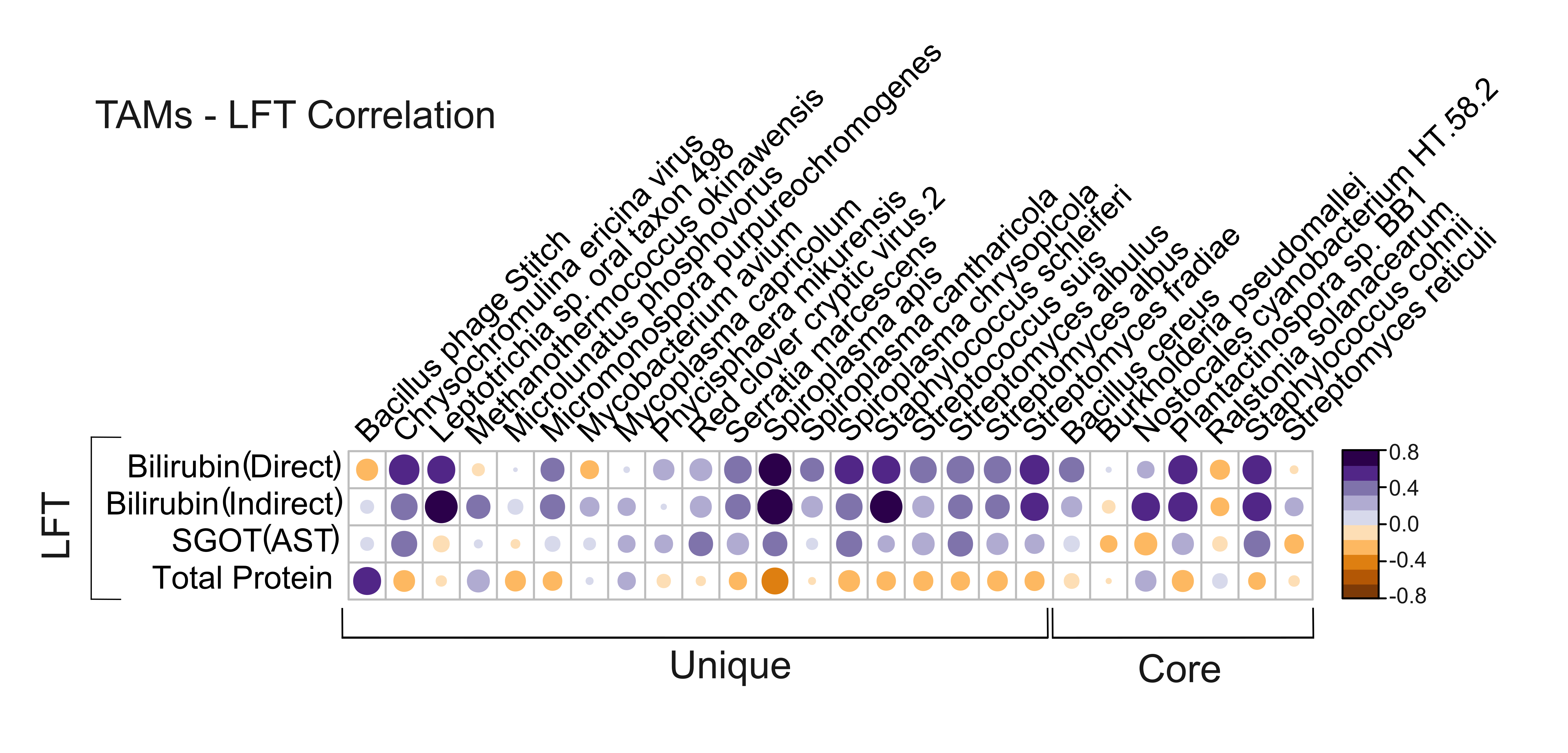

Supplement: S3 Fig — (TIF) [file pntd.0012589.s006.tif]
