# Supplementary material for: Metatranscriptomic insights into the dengue patient blood microbiome: Enhanced microbial diversity and metabolic activity in severe patients
Source: PLoS Negl Trop Dis. 2024 Oct 17;18(10):e0012589. doi: 10.1371/journal.pntd.0012589 (PMC11521311; doi:10.1371/journal.pntd.0012589)
Supplement: Attachment — Submitted filename: Response to Reviewers.docx [file pntd.0012589.s007.docx]

**Response to the Reviewers**

**Dear Editor,**

We take this opportunity to thank you for your time towards facilitating the manuscript peer-review. We thank the anonymous reviewers as well and appreciate the reviewer's feedback.

As we respond to the specific reviewers queries, we would also like to request a subtle change in the title of our manuscript to aptly capture the work.

**Original Title:** "Metagenomic insights into the dengue patient blood microbiome: Enhanced microbial diversity and metabolic activity in severe patients."

**Proposed Title:** "Metatranscriptomic insights into the dengue patient blood microbiome: Enhanced microbial diversity and metabolic activity in severe patients."

**Reason for Change:** Upon further reflection and consideration of the study's methodology, we believe that the term "metatranscriptomic" more accurately represents the focus of our research. The study is based on RNA sequencing data, specifically investigating the transcriptionally active microbes within the blood microbiome of dengue patients. Therefore, "metatranscriptomic" is a more appropriate descriptor than "metagenomic," which traditionally refers to the study of total genetic material without focusing on actively transcribed genes.

**Reviewer #1:**

We would like to thank the Reviewer for the insightful comments and pointing out corrections which we have looked into and made necessary adaptations in the revised submission.

***Methods***

*The study design is robust, and the statistical analyses used are appropriate to support the conclusions. There are no concerns regarding ethical requirements. Although the sample size is limited, it is sufficient to substantiate the conclusions.*

Thank you for the positive feedback to our manuscript. We acknowledge your perspective about the sample size. We hope that in future studies we would be able to respect your opinion.

***Results***

*The analysis presented is well-suited to address the research question, and the results are clearly articulated. The figures are of high quality.*

Thank you for the positive feedback on our analysis and the clarity of our results.

*Minor Comments:*

*•	Table 1: Include dispersion values in the data.*

We have now included dispersion values in the data. The table has now been revised as:

|  | **Dengue Clinical Parameters** | **Mild (n=45)** | **Moderate (n=46)** | **Severe (n=21)** | **p-values** |  |
| --- | --- | --- | --- | --- | --- | --- |
| **Age** | Age | 27 (18–33) | 26 (19–31.75) | 35 (18–43) | 0.14 | b |
| **Gender** | Gender F\|M | 15\|30 | 17\|29 | 4\|17 | 0.34 | a |
| **NS1 Ag** | NS1 antigen | 3.22 (0.83–3.60) | 3.5 (0.86–3.5) | 3.5 (1.9–3.5) | 0.75 | b |
| **CBC** | Total Leucocyte Count (TLC) | 5 (4.6–5.9) | 3 (2.5–3.575) | 2.7 (2.3–3.1) | **< .001** | b |
|  | RBC Count | 4.76 (4.51–4.99) | 4.835 (4.58–5.02) | 4.78 (4.51–5.58) | 0.76 | b |
|  | Packed Cell, Volume | 41.2 (40.1–42.8) | 42.25 (38.4–44.22) | 43.9 (40.4–46.3) | 0.18 | b |
|  | Platelet Count | 175 (155–202) | 160 (150–165) | 125 (112–135) | **< .001** | b |
|  | Neutrophils | 75.9 (66.7–79.5) | 63.3 (58.1–68.65) | 62.6 (57.3–73) | **< .001** | b |
|  | Lymphocytes | 14.1 (9.4–21.3) | 24.5 (20.3–31.975) | 26 (15.8–32.7) | **< .001** | b |
|  | Monocytes | 9.8 (8–12) | 10 (7.85–11.475) | 9 (6.9–11) | 0.32 | b |
|  | Eosinophils | 0.2 (0–0.725) | 0.2 (0.1–0.875) | 0.5 (0.1–1) | 0.32 | b |
|  | Basophils | 0.5 (0.3–0.6) | 0.6 (0.225–0.8) | 0.5 (0.3–0.8) | 0.69 | b |
|  | Hb | 13.6 (12.75–14.3) | 13.9 (12.52–14.5) | 14.1 (13.1–15.3) | 0.28 | b |
| **LFT** | Total Protein | 7.3 (7–7.6) | 6.7 (6.5–7.3) | 6.7 (6.05–7.125) | **0.03** | b |
|  | Albumin | 4.4 (4.05–4.5) | 4 (3.8–4.15) | 4 (3.675–4.1) | **0.05** | b |
|  | Globulin | 3.1 (2.95–3.3) | 3 (2.65–3.15) | 2.65 (2.3–2.925) | 0.06 | b |
|  | A.G. ratio | 1.4 (1.3–1.55) | 1.3 (1.2–1.5) | 1.55 (1.375–1.6) | 0.19 | b |
|  | Bilirubin (Total) | 0.7 (0.55–0.8) | 0.4 (0.3–0.55) | 0.75 (0.5–0.9) | **0.02** | b |
|  | Bilirubin (Direct) | 0.135 (0.12–0.17) | 0.09 (0.07–0.13) | 0.17 (0.13–0.23) | **0.02** | b |
|  | Bilirubin (Indirect) | 0.56 (0.43–0.615) | 0.31 (0.24–0.42) | 0.55 (0.39–0.71) | **0.03** | b |
|  | SGOT- Aspartate Transaminase (AST) | 41 (34.5–76) | 59 (44–80.5) | 97.5 (74.25–141.5) | **0.03** | b |
|  | SGPT- Alanine Transaminase (ALT) | 42 (20–95) | 41 (25.5–59) | 66 (51.75–80.25) | 0.13 | b |
|  | AST/ALT Ratio | 0.93 (0.82–1.725) | 1.48 (1.13–1.95) | 1.705 (1.2–2.17) | 0.08 | b |
|  | Alkaline Phosphatase | 67 (60–89) | 80 (59.5–100) | 82.5 (59.75–124.25) | 0.61 | b |
|  | GGTP (Gamma GT), Serum | 37 (24.5–46.5) | 19 (14–41) | 49 (32–82) | 0.08 | b |

a-chi square; b-kruskal wallis

*•	Table 2: Clarify that these are metabolic pathways.*

We have revised **Table 2** to explicitly clarify that the entries represent metabolic pathways. The last column heading has been changed from “Enriched Pathways” to “Enriched Metabolic Pathways”

*•	Page 25: Italicize "S. marcescens."*

We appreciate the Reviewer for this observation. We have now italicized “*S. marcescens*” in the revised manuscript.

*•	Page 26: Remove italics in "although uncommon in humans."*

Thank you for your attention. We have removed italics in "although uncommon in humans."

***Conclusions***

*The conclusions are well-supported by the data, and the study's limitations are adequately described. The implications for public health are addressed, and the relevance of the work is clearly articulated.*

Thank you for your encouraging feedback on our manuscript.

*Minor Comments:*

*•	Page 26: The statement "these species may serve as microbial markers for evaluating the severity of dengue fever" is significant. I suggest elaborating on this idea, emphasizing the potential for early diagnosis. Additionally, consider mentioning as a limitation that it is unclear whether these bacteria are present at the onset of the disease.*

Thank you for highlighting the significance of the statement regarding the potential of these species as microbial markers for evaluating the severity of dengue fever. We appreciate your suggestion to elaborate on this, and we have expanded the discussion to emphasize the potential for these markers in early diagnosis.

The updated discussion now reads as:

“Given the association of potential pathogens like *B. cereus*, *S. cohnii*, and *B. pseudomallei* with key indicators of dengue severity, these species may serve as microbial markers for evaluating the severity of dengue fever. Detailed characterization and identification of microbial features linked to these infections could facilitate early detection, enabling timely and targeted treatment. Targeting these specific microbes presents a promising approach for early diagnosis, offering a non-invasive and potentially more accurate method for identifying health conditions at their earliest stages. As ours’ is the first study elucidating blood microbiome of Dengue patients with different disease severities, future metatranscriptomic studies in this direction from different cohorts within India as well as globally would be great value addition.”

Additionally, we have incorporated your recommendation to address the limitation concerning the uncertainty of whether these bacteria are present at the onset of the disease.

“Since these studies are rare, the range of microbial dynamics in dengue infection, more so with different disease severity, is just beginning to be explored. One limitation of our study is that it remains unclear whether these microbes are present at the onset of dengue fever. Further research is needed to determine the timing of their appearance/significant differential abundance in relation to the disease's progression, which would be essential for evaluating their potential as early diagnostic markers. Insights would be strengthened with longitudinal studies of the Dengue patients to understand the microbial dynamics. In total, our blood microbiome study represents a preliminary exploration of the early opportunistic TAMs modulating clinical parameters and their enriched metabolic pathways associated with the genes that govern the establishment of severe infection **(Figure 6)**.”

*•	Figure Legend 6: The phrase "active microbial species in the bloodstream exacerbates inflammatory responses and enriched metabolic pathways" implies causation, while the study only shows correlation. The main text does not exhibit this overinterpretation.*

Thank you for pointing out the issue with the wording in **Figure 6** Legend. We acknowledge that the phrase "active microbial species in the bloodstream exacerbates inflammatory responses and enriched metabolic pathways" implies causation, which was not our intention, as the study only demonstrates correlation. We have now revised the figure legend to closely reflect the findings without over-interpreting the data, in line with the main text, and now it reads as:

“This Figure highlights the correlation between the increased presence of specific transcriptionally active microbial species in the bloodstream and the possible exacerbation of inflammatory responses and enriched metabolic pathways, which are associated with enhanced disease severity in the dengue patients.”

***Summary and General Comments***

*This study is highly compelling, linking the blood microbiome of dengue patients with biometric data on the inflammatory response. The use of RNA-seq to confirm that the bacteria are metabolically active is commendable, and the correlations found between certain species and metabolic pathways with disease severity are significant. The study is well-executed, employing appropriate methodologies and statistical analyses.*

*I have only a few minor comments, as noted in the Results and Conclusions sections. It was a pleasure to review this manuscript.*

We are pleased and thankful to receive your thoughtful review.

**Reviewer #2:**

***Summary and General Comments***

*Yadav et al. report the microbial diversity in dengue patients. Dengue is an emerging problem that is associated with climate change and is becoming an increasing problem in India. The study is straightforward and uses a rigorous data analysis pipeline to report on the microbial diversity in clinically relevant specimens. The study is informative, and I only have minor comments that are appended below.*

Thank you for your positive feedback on our study. We are glad that you found our study to be informative, timely and that our data analysis pipeline is robust. We have addressed the minor comments you have provided and incorporated them to further strengthen the manuscript.

*1. Figure 2A – how many patients had overlapping symptoms? It will be useful to represent the data as overlapping Venn Diagrams to illustrate the overlapping and unique symptoms in the patient population.*

Thank you for your suggestion regarding **Figure 2A**. We have now illustrated the overlapping and unique symptoms across the three severity subgroups as Venn Diagram. The text has been included in the revised manuscript under “**Clinical Parameters across the Disease Severities**” result and the figure has been provided as **Supplementary figure (S1 Figure)**. The text reads as:

“Mild symptoms, including fever, chills, vomiting, diarrhoea, headache and nausea, were commonly distributed among all the three severity groups, with additional occurrences of orbital pain, rashes, sore throat, and constipation observed in few cases **(Figure 2a)**. Our data revealed no symptoms exclusive to the severe group, with only a few overlapping symptoms between the mild-moderate and moderate-severe groups. The Venn diagram (**S1 Figure**) visually represents the overlapping and unique symptoms within the patient population.”

*2. How does the data compare to healthy individuals? While the comparison of mild, moderate, and severe cases is very relevant and important, including healthy controls will make the data more meaningful and potentially enable the discovery of Dengue-related biomarkers and microbiome disruption, if any.*

We acknowledge the reviewer query.

In our lab, we are concentrating on understanding the “Modulators of differential disease severities (mild, moderate, severe) and clinical outcome (recovered, mortality) although patients are infected by similar/same pathogen”. Towards that we make use of the clinical data associated with the hospital admitted patients and stratify them into differential disease severities and try understand (using Genomics means of investigation) the factors underlying those differential severities.

In alignment with the research theme, we investigated potential differences in the presence and role of Transcriptionally active microbes (TAMs) between the severity sub-groups of Dengue positive patients (mild, moderate and severe) captured through RNA-Seq and its plausible association with Dengue severity.

In agreement with the Reviewer perspective, in our future study, we plan to compare the severe patients with dengue-negative patients to examine the differences in microbial disruption between severe dengue cases and those without dengue infection.

*3. Please submit all raw data, analysis codes and scripts in a public repository, and include a freely accessible link to the code and data in the manuscript. Eg., GitHub, NCBI.*

Thank you for your suggestion. We would like to inform the reviewer that the raw RNA-seq data has already been uploaded to the NCBI Sequence Read Archive (SRA) under BioProject ID: PRJNA1071729, as mentioned in the manuscript. Additionally, the codes and scripts for the initial metagenomic analysis and taxonomic classification have been published in **Star Protocols (doi:** [**https://doi.org/10.1016/j.xpro.2024.103071**](https://doi.org/10.1016/j.xpro.2024.103071)**)** and now mentioned in the methods section. Further analysis codes are now provided as freely accessible links, and these have been mentioned in the methods section of the revised manuscript:

“The source codes for microbial gene analysis performed in the paper is freely available at <https://github.com/pallawikumari/-Metagenome>.”
